# Genetic diversity of *Culex pipiens* mosquitoes in distinct populations from Europe: contribution of *Cx. quinquefasciatus* in Mediterranean populations

**DOI:** 10.1186/s13071-016-1333-8

**Published:** 2016-01-27

**Authors:** Elena V. Shaikevich, Elena B. Vinogradova, Ali Bouattour, António Paulo Gouveia de Almeida

**Affiliations:** N.I. Vavilov Institute of General Genetics, ul. Gubkina 3, 119991 Moscow, Russia; Zoological Institute, Russian Academy of Sciences, University Embankment 1, 199034 St. Petersburg, Russia; Laboratoire d’Epidémiologie et de Microbiologie Vétérinaire, Service d’Entomologie Médicale, Institut Pasteur de Tunis- Tunis El Manar University, Tunis, Tunisia; Global Health and Tropical Medicine, GHTM, Medical Parasitology Unit, Instituto de Higiene e Medicina Tropical, IHMT, Universidade Nova de Lisboa, UNL, Rua da Junqueira 100, 1349-008 Lisbon, Portugal; Extraordinary professor at ZRU, Department of Medical Virology, Faculty of Health Sciences, University of Pretoria, Pretoria, South Africa

**Keywords:** *Culex pipiens* complex, mtDNA, *COI*, *Wolbachia*, nuclear DNA, hybrid, mitochondrial introgression

## Abstract

**Background:**

Mosquitoes of the *Culex pipiens* complex are cosmopolitan, and important vectors of neglected tropical diseases, such as arbovirosis and lymphatic filariasis. Among the complex taxa, *Cx. pipiens* (with two forms *pipiens* and *molestus*) and *Cx. quinquefasciatus* are the most ubiquitous mosquitoes in temperate and tropical regions respectively. Mosquitoes of this taxa lack of morphological differences between females, but have frank behavioral and physiological differences and have different trophic preferences that influence their vectorial status. Hybridization may change the vectorial capacity of these mosquitoes, increasing vector efficiency and medical importance of resulting hybrids.

**Methods:**

*Culex pipiens* s.l. from 35 distinct populations were investigated by the study of mtDNA, symbiotic bacterium *Wolbachia pipientis*, nuclear DNA and flanking region of microsatellite CQ11 polymorphism using PCR with diagnostic primers, RFLP analysis and sequencing.

**Results:**

Six different mitochondrial haplotypes were revealed by sequencing of the cytochrome oxidase subunit I (*COI*) gene and three different *Wolbachia* (*w*Pip) groups were identified. A strong association was observed between *COI* haplotypes/groups, *w*Pip groups and taxa; haplogroup A and infection with *w*PipII appear to be typical for *Cx. pipiens* form *pipiens*, haplotype D and infection with *w*PipIV for form *molestus*, while haplogroup E, characteristic of *Cx. quinquefasciatus*, were correlated with *w*PipI and found in *Cx. pipiens* sl. from coastal regions of Southern Europe and Mediterranean region. Analysis of microsatellite locus and nuclear DNA revealed hybrids between *Cx. pipiens* form *pipiens* and form *molestus*, as well as between *Cx. pipiens* and *Cx. quinquefasciatus*, in Mediterranean populations, as opposed to Northern Europe. Phylogenetic analysis of *COI* sequences yielded a tree topology that supported the RFLP analysis with significant bootstrap values for haplotype D and haplogroup E.

**Conclusions:**

Molecular identification provides the first evidence of the presence of hybrids between *Cx. quinquefasciatus* and *Cx. pipiens* as well as cytoplasmic introgression of *Cx. quinquefasciatus* into *Cx. pipiens* as a result of hybridization events in coastal regions of Southern Europe and Mediterranean region. Together with observed hybrids between *pipiens* and *molestus* forms, these findings point to the presence of hybrids in these areas, with consequent higher potential for disease transmission.

**Electronic supplementary material:**

The online version of this article (doi:10.1186/s13071-016-1333-8) contains supplementary material, which is available to authorized users.

## Background

Mosquitoes of the *Culex pipiens* complex are important disease vectors with global distribution [1]. Several species, subspecies, and forms are currently recognized as belonging to this complex and are generally considered competent vectors of arboviruses, including West Nile virus and Rift Valley Fever virus, as well as filarial worms and avian *Plasmodia* [2].

Among the complex taxa, *Cx. pipiens* Linnaeus, 1758 and *Cx. quinquefasciatus* Say, 1823 are the most ubiquitous mosquitoes in temperate and tropical regions respectively. *Culex pipiens* sensu stricto L. 1758 is subdivided into three intraspecific forms: “*pallens*” Coquillett 1898, “*molestus*” Forskål 1775 and “*pipiens*” [3, 4]. No consensus exists on the taxonomic status of the members of the complex, with conflicting evidence according to incomplete isolation, hence the existence of hybrid populations, between *Cx. quinquefasciatus* and *Cx. pipiens* s.s. form *pipiens* or form *molestus*, in some contact zones such as North America [5–8], Mexico [9], Argentina [10, 11], the Cape Verde Islands in the Atlantic Ocean, Africa [12], and in Greece (Europe) [13], while exhibiting complete isolation in other regions such as East Africa [14]. Hybrids between other members of *Culex pipiens* complex, such as *Cx. pipiens pallens* and *Cx. pipiens* form *molestus* have been detected in Japan [15].

*Cx. pipiens* includes two forms (or biotypes) denoted as *pipiens* and *molestus* that differ in their physiology and behavior. The *pipiens* form requires a blood meal for egg development (anautogeny), prefers to feed on birds (ornitophylic) and enters into diapause during the winter (heterodynamic). By contrast, the *molestus* form typically lays a first batch of eggs without a blood meal (autogeny), readily feeds on mammals (mamophylic) and remains active yearlong (homodynamic). Remarkably, the *molestus* form commonly adopts underground habitats in colder temperate climate regions and can mate in confined spaces (stenogamy), whereas the *pipiens* form colonizes above-ground habitats exclusively and mates in large, open areas (eurygamy) [1].

The only morphological differences among the members of the *Culex pipiens* complex exist in the genital structure of males. The absence of morphological differences in females and the presence of hybrids make it quite difficult to identify these taxa. Several molecular tools have been developed to differentiate species and forms of the *Cx. pipiens* complex [16–19]. These molecular analyses have also detected recurrent hybridization among *Cx. pipiens* s.l. members where their distribution overlaps, as exemplified by the hybrid populations of *Cx.pipiens* and *Cx.quinquefasciatus* mentioned above. Hybrids between the forms *pipiens* and *molestus* have also been detected in the United States [6], Portugal [20], Netherlands [21], Greece [22] and Morocco [23], although in cases such as the Netherlands, confirmation of these results should be obtained due to possible presence of *Cx. torrentium*. Hybridization in the *Cx. pipiens* complex may change the vectorial capacity of mosquitoes, increasing the vector efficiency of resulting hybrids, which are therefore called bridge vectors [6, 24]. In this context, the analysis of the genetic structure of mosquito populations sheds light on the processes taking place.

Due to the absence of morphological differences between females, molecular tools have been developed to differentiate species and forms, as well as detecting hybridization events. Cytochrome oxidase c subunit I (*COI*) mitochondrial gene has proven to be a reliable marker in the Paleartic region for differentiating among members of the *Culex pipiens* complex [25–27]. Mitochondrial DNA does not recombine, is mostly transmitted through the egg cytoplasm and is often used in phylogenetic studies of insects, including mosquitoes [28–30]. In zones of sympatry, where hybridization occurs between the complex members, nuclear markers are advisable in order to avoid erroneously identifying cases of cytoplasmic introgression [31].

It is known that the symbiotic bacterium *Wolbachia* manipulates reproduction of *Culex pipiens* complex mosquitoes by cytoplasmic incompatibility, a form of embryonic lethality, between infected males and uninfected females or between individuals carrying incompatible strains. This can potentially result in reproductive isolation between host populations. Five distinct *Wolbachia* groups (*w*Pip), that are closely related evolutionarily, have been documented in mosquito’s complex *Culex pipiens* [32]. These *w*Pip groups show different incompatibility status [33, 34]. *Wolbachia* and host mitochondria are co-transmitted in the egg cytoplasm, constituting valuable markers, and the association between *w*Pip and mtDNA groups was determined [32]. A recent study [35] found that cytoplasmic introgression could be mediated by the maternally-inherited bacterium *Wolbachia pipientis*: mtDNA and *w*Pip are associated with regular co-transmissions between *Cx. pipiens* members through hybridization events across the complex. Moreover, conflicting evidence has been given regarding the interference of the *Wolbachia* symbionts on the vector competence to arboviruses [36, 37].

The aim of this work was the analysis of the genetic diversity of mosquito populations and to detect hybridization events that might shed light on the contribution of *Cx. quinquefasciatus* and *Cx.pipiens* form *pipiens* and form *molestus* in genetic diversity of European and Mediterranean populations by analyzing nuclear DNA markers, *COI* gene mtDNA polymorphism and its association with *w*Pip infection.

## Methods

### Mosquito samples

Mosquitoes of the *Culex pipiens* complex, adults and larvae, sampled from 2007 to 2012, originating from urban and suburban sites, and from laboratory colonies were analyzed. Geographical origins ranged from Eastern to Western, as well as, Northern to Southern, Europe, but also samples from Morocco, Tunisia, Israel and India (Table 1). The collection comprised the two main members of the complex, *Cx.quinquefasciatus* and *Cx. pipiens* with the two forms *pipiens* and *molestus* of which 225 individuals from 20 populations were studied for mtDNA diversity for the first time, and 355 samples from 15 populations that had been studied earlier [13, 38, 39], yielding a total of 580 specimens from 35 populations. Thus, all 580 specimens were processed for discrimination at taxa level and typed at *COI* locus haplotype (Table 1), whilst a subset of 274 samples were studied at *w*Pip and nuclear loci (Table 3), and 24 were fully sequenced *de novo* for *COI* mtDNA and analyzed for phylogenetic relationship jointly with another 24 sequences previously obtained [40].

**Table 1:** Data on *Culex pipiens* populations and results of RFLP analysis of 5′*COI* gene

Origin (Country and locality)	*COI* type	Map legend (Fig. 3)	Coordinates latitude/longitude	Stage of development, sampling site	*Cx. pipiens* taxa	Number	Supplied by	*COI* type reference
Russia, Moscow region (Iksha, Luzki)	A/B/C	1	56°09′N 37°31′ E	larvae, rural, above ground	*Cx. pipiens* f. pipiens*	47	M. Fedorova	[38]
Russia, N. Novgorod region	A/B/C	2	55°02′N 43°15′E	larvae, rural, above ground	*Cx. pipiens*f. *pipiens*	10	E. Vinogradova	[38]
Russia, Krasnodar	A/B/C	3	45°02′N 38°58′E	larvae, suburban, above ground	*Cx. pipiens*f. *pipiens*	28	E. Vinogradova	[38]
Russia, Volgograd (Liteishik,Sarpinsky)	A/B/C	4	48°42′N 44°31′E	larvae, rural, above ground	*Cx. pipiens*f. pipiens*	20	M. Fedorova	[38]
Russia, North Kaukas	A/B/C	5	43°29′N 43°37′E	larvae, suburban, above ground	*Cx. pipiens*f. *pipiens*	28	E. Vinogradova	[38]
Germany, Hannover	A/B/C	6	52°22′N 09°43′E	larvae, urban, above ground	*Cx. pipiens*f. *pipiens*	17	E. Shaikevich	This study
Germany, Berlin	A/B/C	7	52°31′N 13°23′E	larvae, urban, above ground	*Cx. pipiens*f. *pipiens*	9	E. Shaikevich	This study
France, Prades-le-Lez1	A/B/C	8	43°42′N 03°52′ E	larvae, above ground	hybrid^a^	17	O. Duron	This study
France, Prades-le-Lez2	A/B/C	9	43°42′N 03°52′ E	larvae, above ground	hybrid^a^	22	O. Duron	This study
France, St-Nazaire de Pezan	A/B/C	10	43°38′N 04°08′ E	larvae, above ground	hybrid^a^	12§	O. Duron	This study
France,T7 strain,Montpellier	A/B/C	11	43 36′N 03°52′E	larvae, lab culture	*Cx. pipiens*f. *molestus*	11	O. Duron	This study
Morocco, Casablanca	A/B/C	12	33°32′N 07°35′W	imago, suburban, above ground	hybrid^a^	2	A.- B. Failloux	This study
Russia, Moscow	D	13	55°45′N 37°37′E	larvae, urban, underground	*Cx. pipiens*f. molestus*	21	M. Fedorova	[38]
Russia, St-Petersburg	D	14	59°57′N 30°18′E	larvae, urban, underground	*Cx. pipiens*f. molestus*	22	E. Vinogradova	[38]
Russia, N. Novgorod	D	15	56°20′N 44°00′E	larvae, urban, underground	*Cx. pipiens*f. molestus*	10	E. Vinogradova	[38]
Russia, Krasnodar	D	16	45°02′N 38°58′E	larvae, urban, above ground	*Cx. pipiens*f. molestus*	52	E. Vinogradova	[38]
Russia, Tomsk	D	17	56°30′N 84°58′E	larvae, urban, underground	*Cx. pipiens*f. *molestus*	10	A.Sibataev	[38]
Russia, Ekaterinburg	D	18	56°53′N 60°35′E	larvae, urban, underground	*Cx. pipiens*f. *molestus*	24	N.Nikolaeva	[38]
Russia, Petrozavodsk	D	19	62°47′ N 34°20′E	larvae, urban, underground	*Cx. pipiens*f. molestus*	10	S.Karpova	[38]
Russia, Volgograd	D	20	48°42′N 44°31′E	larvae, urban, underground	*Cx. pipiens*f. molestus*	30	M. Fedorova	[38]
Russia, Vladikavkaz	D	21	43°01′N 44°39′E	larvae, urban, underground	*Cx. pipiens*f. *molestus*	1	E. Vinogradova	[38]
Germany, Berlin	D	22	52°31′N 13°23′E	imago, urban, indoor space	*Cx. pipiens*f. *molestus*	4	E. Shaikevich	This study
Germany, Hannover	D	23	52°22′N 9°43′E	imago, urban, indoor space	*Cx. pipiens* f. *molestus*	1	E. Shaikevich	This study
Italy, Piedmont (Frugarolo, Tortona)	D	24	44°54′ N 8°37′ E	imago and larvae, urban, above ground	hybrid^a^	18¥	A. Talbalaghi	[39]
Tunisia, Nefza	D	25	37°06′N 9°11′E	imago and larvae, above ground	hybrid^a^	16†	A. Bouattour	This study
Tunisia, Tabarka	D	26	36°57′N 8°45′E﻿	imago and larvae, above ground	hybrid^a^	12†	A. Bouattour	This study
Morocco, Casablanca	D	27	33°32′N 7°35′W	imago, suburban, above ground	hybrid^a^	1	A.- B. Failloux	This study
India, Hydarabad	E/E1	28	17°8′ N 78° 31′ E	larvae, lab culture	*Cx. quinquefasciatus*	20	E. Vinogradova	This study
India, Pondicherry	E/E1	29	12°25′ N 80°41′ E	larvae, lab culture	*Cx. quinquefasciatus*	23	E. Vinogradova	This study
Portugal, Comporta	E/E1	30	38°22′ N 8°46′ W	imago, above ground	*Cx. pipiens*f. *pipiens*	6	P. Almeida	This study
Portugal, Comporta	E/E1	30	38°22′ N 8°46′ W	imago, above ground	*Cx. pipiens*f. molestus*	14	P. Almeida	This study
Italy, Viterbo	E/E1	31	42°23′ N 12°7′ E	larvae, urban, above ground	*Cx. pipiens* s.l.	15	E. Vinogradova	This study
Israel, Haifa	E/E1	32	32° 49′N34° 57′E	imago, urban, indoor space	*Cx. pipiens* s.l.	7	E. Shaikevich	This study
Morocco, Tanger	E/E1	33	35° 46′ N 5° 48′ W	imago, urban, above ground	pip/quin hybrid^b^	13	A.- B. Failloux	This study
Greece, Cyprus	E/E1	34	34°46′N 32°25′E	imago, urban, indoor space	hybrid^a^	3§	E. Vinogradova	This study
Greece, Kos	E/E1	35	36°49′N 27°06′E	imago, urban, indoor space	pip/quin hybrid^b^	24	E. Vinogradova	[13]
					Total	580		

*Cx. pipiens* s.l. correspopnds to unknown taxa discrimination

*-expression of autogeny was studied, hybrid^a^ – pipiens/molestus hybrid populations according to CQ11 assay, pip/quin hybrid^b^ - according to ACE2 assay see Table 4

§ - 1 with unknown status; ¥ - 6 with unknown status; † − 3 with unknown status

### *Culex pipiens* taxa discrimination

*Culex quinquefasciatus* and *Cx. pipiens* were discriminated using a specific PCR assay based on the acetylcholinesterase-2 gene (ACE2-assay) [18]. Identification of the form *molestus* and the form *pipiens* of *Cx. pipiens* was made on the basis of the ovarian status of females (autogeny) and genetically by both CQ11 [19] and *COI* [25] assays. The expression of autogeny was studied in the laboratory, for most underground and above ground populations from Russia, insectary colony T7 from France, and in samples from Portugal by the respective collectors (Table 1). Autogeny of *Cx. pipiens* from the other populations was not studied. Individuals whose autogeny status had not been determined, and that came from collections which included either *pipiens* or *molestus*, as well as hybrid forms according to CQ11 analysis, were denominated as *Cx. pipiens* “hybrid” and those to whom neither autogeny determination nor CQ11 assay had been performed or the results of CQ11 were inconclusive, were denominated as “unknown” in Table 1.

### Molecular typing

DNA was extracted from mosquitoes preserved in ethanol using the DIAtom™ DNA Prep Kit (Isogen, Moscow, Russia). Polymerase chain reactions were run in thermocyclers GeneAmpR PCR System 2700 (Applied Biosystems, Foster City, CA, USA) with amplification kits GenePak™ PCR Core (Isogene, Moscow, Russia).

### Mitochondrial DNA typing

The DNA sequences of the *COI* mtDNA gene of 1150 bp were amplified using the TY-J-1460 [41] and UEA10 primers [42] as previously described [40]. Twenty four sequences were obtained *de novo* from an ABI 310 automated sequencer using the ABI PRISM BigDye Terminator Cycle Sequencing kit (Applied Biosystems, Foster City, CA, USA) for mosquitoes originated from Russia (9 sample sites), Germany (2 sample sites), Italy (2 sample sites), Greece (1 sample site), Portugal (1 sample site), Tunisia (2 sample sites), Israel (1 sample site) (Table 2). Sequences were analyzed using Chromas software (http://www.technelysium.com.au). Six different haplotype sequences, which we denoted as A, B, C, D, E [40] and another found in this work for the first time, E1, have been deposited in GenBank under numbers KM233145-KM233150, as a result of this work.

**Table 2:** Distribution of *COI* haplotypes between *Cx. pipiens* taxa base on sequence analysis

Populations (country, name)		Taxonomy status	Frequency of mt haplotype (number of specimens)					
			A	B	C	D	E	E1
Russia	Moscow region, Iksha	*Cx.pipiens* f.*pipiens*	0.4 (2)	-	0.6 (3)	-	-	-
	Moscow region, Luzki	*Cx.pipiens* f.*pipiens*	0.3 (1)	0.7 (2)	-	-	-	-
	Volgograd Region, Sarepta	*Cx.pipiens* f.*pipiens*	1 (1)	-	-	-	-	-
	Volgograd Region, Liteishik	*Cx.pipiens* f.*pipiens*	-	-	1 (1)	-	-	-
	Petrozavodsk	*Cx.pipiens* f.*molestus*	-	-	-	1 (1)	-	-
	Saint Petersburg	*Cx.pipiens* f.*molestus*	-	-	-	1 (2)	-	-
	Moscow	*Cx.pipiens* f.*molestus*	-	-	-	1 (1)	-	-
	Nizhniy Novgorod	*Cx.pipiens* f.*molestus*	-	-	-	1 (1)	-	-
	Volgograd	*Cx.pipiens* f.*molestus*	-	-	-	1 (5)	-	-
Germany	Berlin	*Cx.pipiens* f.*pipiens*	0.5 (1)	-	0.5 (1)	-	-	-
		*Cx.pipiens* f.*molestus*	-	-	-	1 (2)	-	-
	Hannover	*Cx.pipiens* f.*pipiens*	0.5 (1)	-	0.5 (1)	-	-	-
		*Cx.pipiens* f.*molestus*	-	-	-	1 (1)	-	-
Italy	Piedmont	*Cx.pipiens* hybrid^a^	-	-	-	1 (2)	-	-
	Viterbo	*Cx.pipiens* s.l.	-	-	-	-	1 (1)	-
Portugal	Comporta	*Cx.pipiens* f.*pipiens*	-	-	-	-	1 (3)	-
	Comporta	*Cx.pipiens* f.*molestus*	-	-	-	-	0.14 (1)	0.86 (6)
Greece	Cyprus	*Cx. pipiens* hybrid^a^	-	-	-	-	1 (1)	-
Israel	Haifa	*Cx. pipiens* s.l.	-	-	-	-	1 (2)	-
Tunisia	Nefza	*Cx. pipiens* hybrid^a^	-	-	-	1 (5)	-	-
	Tabarka	*Cx. pipiens* hybrid^a^	-	-	-	1 (2)	-	-
India	Hydarabad	*Cx. quinquefasciatus*	-	-	-	-	1 (2)	-
	Pondicherry	*Cx. quinquefasciatus*	-	-	-	-	1 (2)	-

^a^based on CQ11 assay, with no data regarding autogeny status

These sequences were compared with 24 previously studied full-size DNA sequence of the *COI* gene (1548 bp) of both forms of *Cx.pipiens* originated from 10 geographically distinct sample sites from Russia (Gene Bank accession numbers FN395171-FN395190) and *Cx. quinquefasciatus* originated from two sample sites from India (FN395201- FN395204) [40].

**Fig. 1:**
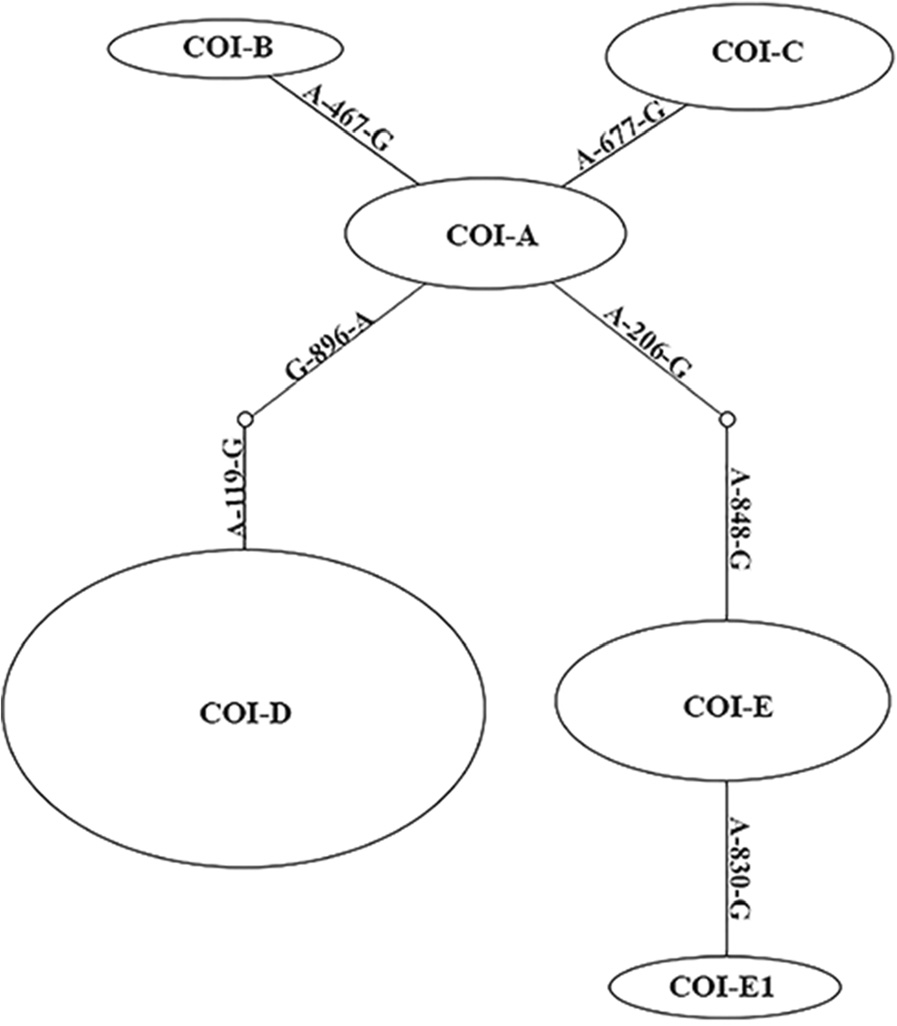
Network analysis based on statistical parsimony showing the relationships of the *Cx. pipiens COI* haplotypes. Mutations are shown on the branches. The size of the ovals are proportional to the number of the occurring haplotypes in 48 samples from 20 localities (Table 2)

*Culex* mtDNA of 580 specimens from 35 geographical populations was also genotyped using a series of specific PCR-RFLP (restriction fragment length polymorphism) assays based on the DNA variability of *COI*gene [25, 43]. The 5′ region of the *COI* gene of 603 bp was amplified using primers CulexCOIF and CulexCOIR [25]. PCR conditions were the following: primary denaturing – 5 min at 94°С; 35 cycles: denaturing at 94°С – 30 s, annealing at 55°С – 30 s, synthesis at 72°С – 40 s; final synthesis at 72°С – 10 min. *Hae*III digestion of the *COI* PCR products allowed the discrimination of D haplotype from A, B, C and E haplotypes [25, 43]. However, *Hae*III has no recognition site for GG’CC on the *COI* sequence of type D and PCR-product of amplification remains unchanged, 603 bp, whereas the *COI* fragment of other types resulted in two fragments - 397 and 206 bp respectively (Additional file 1A) - after restriction with *Hae*III. After digestion with *Alu*I, the *COI* PCR-products of A, B, C and D types resulted in 8 fragments (189, 171, 99, 67, 45, 15, 12, 5 bp), 5 of which are visible by electrophoresis in a 2 % agarose gel. The *COI* fragment of type E and E1 is cut into 7 fragments (189, 171, 144, 67, 15, 12, 5 bp) only because the mutation at position 206 (Fig. 1) inactivates the *Alu*I restriction site (AG’CT). Consequently, the 144 bp fragment is diagnostic for haplotypes E and E1 allowing their discrimination (Additional file 1B). Reactions were carried out in a restriction mixture consisting of 5 μl *COI* PCR product, 0.2 μl (2 units) of the enzyme, 3 μl buffer, 0.3 μl BSA and 21.5 μl ddH2O. Both *Hae*III and *Alu*I restriction mixtures were incubated for 2 hours at 37 °C. At least two technical replicates were performed. Results were visualized by electrophoresis in a 2 % agarose gel. As it is not known whether or not the insectary lines descended from one or more female founders, mtDNA polymorphisms were studied for 10–20 individuals per population.

### Nuclear DNA typing

Complex species and form identification, using ACE2 (with primers B1246s, ACEpip and ACEquin) and CQ11 assays, respectively were performed as described by authors [18, 19]. After amplification with primers B1246s, ACEpip and ACEquin, PCR product of 610 bp is characteristic for *Cx. pipiens* and of 274 bp for *Cx. quinquefasciatus* [18]. The CQ11 PCR-product approximately 200 bp in size is characteristic for *Cx. pipiens* form *pipiens* and 250 bp for form *molestus*. However, CQ11 amplicons from *Cx. quinquefasciatus* yields a PCR product of 250 bp too; therefore the authors recommend the use of a combination of both tests, CQ11 and ACE2 in areas of sympatry of the two species [19].

### ACE2 sequencing

For amplification of *ACE*2 gene of samples from Kos we used the primers F1457 5′–GAGGAGATGTGGAATCCCAA–3′ and B1246 5′–TGGAGCCTCCTCTTCACGGC–3′ and PCR conditions described earlier [16]. Amplicons of the *ACE*2 gene were approximately 710 bp. PCR products were excised from a 1 % agarose gel and purified using a QIAquick Gel Extraction kit (Qiagen, USA) according to the manufacturer’s instructions. The PCR products from two mosquito were cloned using the kit pGEM-T Easy Vector Systems (Promega, USA). The clones were screened for the presence of different *ACE*2 alleles by PCR-RFLP test: the restriction enzyme *Sau*3AI (Fermentas) cuts the “*pipiens”* allele into three fragments (330 bp, 213 bp and 167 bp), and the “*quinquefasciatus”* allele into two fragments (543 bp and 167 bp). Bacterial cells suspension after denaturation in boiling water bath used as a DNA template in PCR with F1457 and B1246 primers and 10 μL of the PCR product after amplification were digested with 2 units of enzyme for 2 hours at 37 °C. PCR products from individual bacterial clones were sequenced from both fragment’s ends using the equipment ABI PRISM 310 and the BigDye Termination kit (Applied Biosystems, USA), according to the manufacturer’s instructions. Sequences were analyzed using Chromas software (http://www.technelysium.com.au) and two different alleles of *ACE*2 gene were deposited in GenBank under numbers KU163609-10.

### *Wolbachia* infection typing

The *w*Pip infections were genotyped *de novo* in a subsample of 274 individuals representative of all *COI* haplotypes and assigned to one group (*w*Pip-I to *w*Pip-V) using PCR-RFLP assays based on two *Wolbachia pipientis* markers, *ank2* and *pk1,* as previously described [30]. PCR-products about 310 and 510 bp were obtained after *ank*2 amplification. Specific *pk1* PCR-products were approximately 1350 bp in size. The digestion with endonuclease *Hinf*I of the *ank2* PCR products provided three alleles: а (313 bp), b (217, 195, 98 bp) and с (293, 217 bp). After digestion of the *pk1* PCR products with endonuclease *Taq*I, three alleles were obtained: a/e (903, 430 bp), c (851, 498 bp) and d (497, 251, 107 bp). The alleles a and e of *pk1* gene have the same fragment sizes and therefore needed additional treatment of the *pk1* PCR products by restriction endonuclease *Pst*I, resulting in two alleles: a (903, 303, 141 bp) and e (903, 430 bp). After this additional digestion with *Pst*I, alleles a, c and d of the pk1 gene were obtained. According to Atyame and coauthors (2011) [32], different alleles of *pk1* and *ank2* genes correspond to one of five groups, *w*Pip-I to *w*Pip-V.

### Data analysis

Forty-eight *COI* sequences, 1150 bp, the origin of which is shown in Table 2, were analyzed using the software Chromas (http://www.technelysium.com.au) and aligned and analyzed using MEGA v. 6.0 [44].

Mitochondrial *COI* haplotype network analysis was performed for 48 sequences using statistical parsimony with the program TCS v.1.21 [45]. The network connection limit was set at 95 %. The resulting networks identify both the relationship between the different haplotypes as well as the number of substitution connecting haplotypes.

The evolutionary history was inferred by using the Maximum Likelihood method based on the Hasegawa-Kishino-Yano model [46]. The tree with the highest log likelihood (−1539.7457) is shown. Initial tree(s) for the heuristic search were obtained by applying the Neighbor-Joining method to a matrix of pairwise distances estimated using the Maximum Composite Likelihood (MCL) approach. The tree was drawn to scale, with branch lengths measured in the number of substitutions per site. Bootstrap coefficients were calculated for a 1000 repeats. All positions containing gaps and missing data were eliminated.

Chi-square for association between *COI* haplotype and mosquito taxa was tested with GraphPad InStat (www.graphpad.com accessed in May 2014) based on the RLFP analysis of 544 samples, specimens for which the taxonomic status was not determined, i.e. 36 specimens denoted as “unknown” (Table 1), were excluded from the analysis.

## Results

### Polymorphism of the DNA sequence of the mitochondrial gene *COI*

Based on the differences in the nucleotide composition of the gene *COI*, we found 6 mitochondrial haplotypes in mosquitoes of the *Culex pipiens* complex, denoted as A, B, C, D, E, and a new one found in this work, E1. These haplotypes vary in seven substitutions (Fig. 1). Haplotype D is characterized by two fixed substitutions in positions 119 and 896 when compared with A, B, C or E and E1 types. By an additional single mutation haplotype B (in position 467) and haplotype C (in position 677) differ from haplotype A. Haplotypes E and E1 differ from A, B, C and D in positions 206 and 848, while haplotype E1 differs from E, by a single additional mutation in position 830.

Six sequences from Portuguese mosquitoes were only sequenced in the second half of the gene (one sequence with 486 bp and the other five with *ca*. 705–784 bp), which however, included the site where haplotypes E and E1 differ. These were 4 *molestus* with haplotype E1, 1 *pipiens* and 1 *molestus* with haplotype E. So, we temptatively included them into analysis, for a total of 54 sequences (Table 2). Among the 54 samples investigated, 6 specimens had haplotype A, 2 - haplotype B, 6 - haplotype C, 22 - haplotype D, 12 - haplotype E and 6 - haplotype E1 (Table 2). Haplotypes A and C were found in samples from Russia and Germany. Haplotype B was found in one population only: Luzki, from Russia. Haplotype D was found in Russia, Germany, northern Italy (Piedmont), Tunisia and Morocco. Haplotype E was found in India, Italy (Viterbo), Israel and Greece (Cyprus). In Portugal, two haplotypes were found: E and E1.

### PCR-RFLP assays of the *COI* gene

The differences in the nucleotide composition of the 5′ region of the *COI* gene made it possible to choose restriction endonucleases for PCR-RFLP assay [25, 43]. Characteristic 603 bp amplification products were obtained for all *Culex* spp. mosquitoes being studied. A first assay using *Hae*III restriction endonuclease made it possible to identify *COI* type D. Two hundred and 32 mosquitoes with haplotype D were all originated from underground sampling sites from Russia, indoor sites from Germany and also open habitats from Italy, Tunisia and Morocco.

The second assay using *Alu*I restriction endonuclease made it possible to identify E and E1 haplotypes. Sequences of E and E1 types cannot be distinguished using the PCR-RFLP method. Haplotypes E (E1) were found in 125 of the specimens being examined: mosquitoes from Italy, Portugal, Greece, Israel, Morocco and India.

Haplotypes A, B and C cannot be differentiated using the PCR-RFLP approach, so we labeled them as of A (B, C) in Tables 1, 3 and 4. These haplotypes were found in 223 mosquitoes from open habitats from Russia, Germany, France and Morocco and in laboratory line T7. Altogether, 580 individuals from 35 populations were further tested by PCR-RFLP (Table 1).

**Table 3:** The association between mtDNA, type of bacteria *Wolbachia* and ACE2 and CQ11 nuclear loci

Sampling site	Number	*COI* type	*w*Pip type	ACE2^b^type			CQ11 results ^c^				References
				pip	quin	hybrid	pip	mol/quin	hybrid	neg	
Russia, Moscow region	7	A,B,C	II	7	-	-	7	-	-	-	This study
Germany, Berlin	9	A,C	II	9	-	-	9	-	-	-	This study
Germany, Hannover	17	A (B,C)	II	17	-	-	17	-	-	-	This study
Russia, Volgograd	12	A (B,C)	II	12	-	-	12	-	-	-	This study
France, Prades-le-Lez 1	16	A (B,C)	II	16	-	-	14	-	2	-	This study
France, Prades-le-Lez 2	22	A (B,C)	II	22	-	-	16	3	3	-	This study
France, Saint-Nazaire de Pezan	12	A (B,C)	II	12	-	-	9	-	2	1	This study
T7 strain, France,Montpellier	11	A (B,C)	II	11	-	-	-	8	1	-	This study
Morocco, Casablanca	2	A (B,C)	II	2	-	-	-	-	2	-	This study
Russia, S-Peterburg1^a^	8	D	IV	8	-	-	-	6	2	-	This study
Russia, S-Peterburg2^a^	7	D	IV	7	-	-	-	3	4	-	This study
Russia, Tomsk^a^	9	D	IV	9	-	-	ND	ND	ND	ND	[35]
Russia, Ekaterinburg^a^	6	D	IV	6	-	-	ND	ND	ND	ND	[35]
Russia, Moscow^a^	20	D	IV	20	-	-	-	18	2	-	This study
Germany, Berlin^a^	4	D	IV	4	-	-	-	3	1	-	This study
Germany, Hannover^a^	1	D	IV	1	-	-	-	1	-	-	This study
Russia, Volgograd^a^	8	D	IV	8	-	-	-	5	3	-	This study
Italy, Piedmont	18	D	IV	18	-	-	9	-	3	6	This study
Tunisia, Nefza	16	D	IV	16	-	-	7	4	2	3	This study
Tunisia, Tabarka	12	D	IV	12	-	-	4	2	3	3	This study
Morocco, Casablanca	1	D	IV	1	-	-	-	-	1	-	This study
Portugal, Comporta	4	E	I	4	-	-	3	1	-	-	This study and CQ11 from [20]
Portugal, Comporta	6	E1	I	6	-	-	1	4	1	-	
Greece, Kos	24	E	I	11	-	13	3	7	14	-	This study and ACE2 and CQ11 from [13]
Greece, Cyprus	3	E	ND	3	-	-	-	-	2	1	This study
Morocco, Tanger	13	E	I	6	-	7	3	-	10	-	This study
India, Pondisherry	6	E	I	-	6	-	ND	ND	ND	ND	This study
Total	274										

*pip* pipiens, *mol* molestus, *quin* quinquefasciatus

*ND* not determined

^a^undergound (or indoor) sampling sites

^b^ ACE2 assay: *Cx. pipiens* (both forms) - 610 bp, *Cx. quinquefasciatus* - 274 bp, hybrid - 610 and 274 bp

^c^ CQ11 assay: *Cx. pipiens* f. *pipiens* - 200 bp, *Cx. pipiens* f. *molestus*/*Cx. quinquefasciatus* - 250 bp, hybrid - 250 and 200 bp, neg - PCR is negative

**Table 4:** Distribution of *COI* haplotypes between *Cx. pipiens* taxa base on PCR-RFLP

*COI* type	*Cx. pipiens* taxa					N (544)
	*Cx. pipiens* f. pipiens	*Cx. pipiens* f. molestus	*Cx. quinquefasciatus*	*Cx. pipiens*/*Cx. quinquefasciatus* hybrid	*Cx. pipiens*/*molestus* hybrid	
Group A	201 (117 + 84^a^) (91 %)	11^a^ (5 %)	0	0	10^a^ (4 %)	222
D	20^a^ (9 %)	179 (137 + 42^a^) (81 %)	0	0	21^a^ (10 %)	220
Group E	9^a^ (9 %)	14^a^ (14 %)	43 (42 %)	33 (32 %)	3^a^ (3 %)	102

Percentages were calculated along respective line, among 544 individuals, excluding inconclusive from the analysis. Chi-square = 732.71, d.f. = 8, *P* < 0.0001

^a^based on CQ11

### Nuclear locus analysis

Since in some cases inconsistencies between the taxonomic status of *Cx. pipiens* and expected type of *COI* (as with French T7 laboratory line or with Portuguese mosquitoes) have been detected (Table 1), we tested nuclear DNA polymorphism of such markers as ACE2 [18] and microsatellite marker CQ11 [19] to clarify the taxonomic status and to reveal possible cases of hybridization. ACE2 and CQ11 assays could not be performed for all studied individuals, namely from Israel and Viterbo, Italy, due to limitation of DNA availability and budget constraints. In some populations, taxa were defined according to known and previously verified autogeny (namely Russian and Portuguese) and known lab line origin (Indian), of which CQ11 was determined in a subsample of 274 specimens (Table 3).

The ACE2 marker allows for differentiation between *Cx. pipiens* (without separation into forms) and *Cx. quinquefasciatus*. After the amplification with the primers B1246s, ACEpip and ACEquin the majority of mosquito samples yielded the PCR product of 610 bp, characteristic for *Cx. pipiens* (Table 3). The exceptions were specimens collected on the Greek island of Kos (13 of the 24 samples) [13], and specimens from Tanger, Morocco (7 of the 13 samples) in which we found specific PCR products for *Cx. pipiens* (610 bp) and for *Cx. quinquefasciatus* (274 bp), i.e. these samples corresponded to hybrids between these taxa (Additional file 2).

The test based on polymorphism on the flanking region of the microsatellite locus CQ11 has been designed to identify both forms of *Cx. pipiens* (form *pipiens* and form *molestus*) and their hybrids. However, the same size PCR-product is obtained for *Cx. quinquefasciatus* and *Cx. pipiens* form *molestus*. Therefore, we took into account the results of both methods, ACE2 and CQ11 as is recommended by authors [19]. In Tanger, Morocco, 6 samples were *Cx. pipiens*/*Cx. quinquefasciatus* hybrid and 2 samples were *Cx. pipiens* form *pipiens*, according to both assays, 4 samples were *Cx. pipiens* by ACE2 and hybrid by CQ11 and 1 sample was *Cx. pipiens*/*Cx. quinquefasciatus* hybrid by ACE2 and *Cx. pipiens* form *pipiens* by CQ11 PCR-results (Additional file 2). The discrepancy in the results of the analysis based on ACE and CQ11 loci were also obtained in samples from Kos, Greece [13]. Such cases indicate recombination processes in the hybrid population and CQ11 hybrids from these collections are likely to be regarded as hybrids between *Cx. pipiens* and *Cx. quinquefasciatus* rather than hybrids between *pipiens* and *molestus* (11 samples in Tanger and 22 samples in Kos, total 33 in Table 4).

In order to ascertain these PCR results, a larger part of *ACE*2 gene was cloned and sequenced. Particularly the region in which ACE marker is included, namely part of exon 2, intron 2 and part of exon 3, as described in [16]. Analysis of nucleotide sequences confirmed the occurrence of two alleles of the *ACE*2 gene in one individual mosquito (alignment shown in Additional file 3). Following Blast analysis, one allele of *ACE*2 gene of sample Kos1 in our study was completely similar to *Cx. pipiens ACE2* gene sequences (Accession No. AY196910), while the second allele of *ACE*2 gene of the same sample Kos1 was similar to sequence of this gene from *Cx. quinquefasciatus*, 99 % identity with all published sequences in GenBank (for example, Accession No. AY196911), confirming PCR results suggestive of “*pipiens*/*quinquefasciatus*” hybrid.

According to the CQ11 analysis of 274 specimens, hybrids between *pipiens* and *molestus* were detected in almost all samples collected in the Mediterranean region, irrespective of the type of cytoplasmic structures (Table 3). Hybrids between *pipiens* and *molestus* determined by this method were also found earlier in populations from Portugal [20] and Morocco [23], from which some specimens examined in this work were taken.

### Typing of *Wolbachia* polymorphism

The association between *COI* and symbiotic intracellular bacterium *Wolbachia pipientis* was studied in 274 *Cx. pipiens* sl. individuals representative of all *COI* haplotypes (Table 3). Infection with *Wolbachia* was detected in all specimens examined and specific *ank2* and *pk1* PCR-products were observed. In our collection we found the three known *ank2* alleles (a, b and c) and the three known *pk1* (a, c and d) alleles (Additional file 4). Using specific PCR-RFLP assays enabled us to genotype and assign the *w*Pip infections of each specimen to a group, from *w*Pip-I to *w*Pip-V. All individuals with mtDNA haplotypes A, B or C appeared to be infected with *w*Pip-II whereas those with mtDNA haplotype D with *w*PipIV, and those with mtDNA haplotype E and E1 with *w*PipI (Table 3).

Based on the fact that the haplotypes A, B and C are close, suggesting that haplotypes B and C are derived from haplotype A (Fig. 1) and also on the fact that these haplotypes are transmitted in *Cx. pipiens* together with the bacterium *w*Pip-II, we have combined them into a group of mitochondrial haplotypes here denominated - haplogroup A. Similarly, haplotype E1 is likely derived from haplotype E and both are transmitted in association with the *w*PipI infection, so we named them haplogroup E. The only other haplotype we detected was haplotype D, which is linked with *w*PipIV infection. Thus, we have identified three groups of mitochondrial haplotypes of *COI* gene that are associated, respectively, with three groups of symbiotic bacteria in *Culex pipiens* complex mosquitoes, similar with polymorphism of *W. pipientis* and groups of mitochondrial haplotypes of cytB [35].

A highly statistically significant correlation was observed between *COI* type and Taxa (Table 4). Haplogroup A (A, B, C) and *w*PipII was found in 201*Cx. pipiens* form *pipiens* specimens, 11*Cx. pipiens* form *molestus* and 10 hybrid based on CQ11 assay from southern France and Morocco, and 1 sample from France, (St-Nazaire de Pezan) with negative CQ11 results, the so called 0 allel, denoted as “unknown” in Table 1. Haplotype D and *w*PipIV was found in 179 *Cx.pipiens* form *molestus* from northern European counties and in 20 *Cx.pipiens* form *pipiens* specimens, 21 hybrid and in 12 specimens with “unknown” status from Italy, Piedmont and Tunisia. Haplogroup E (E, E1) and *w*PipI was found in 43 *Cx.quinquefasciatus* samples, 33 *Cx. pipiens*/*Cx. quinquefasciatus* hybrids, 9 *Cx. pipiens* form *pipiens* and 14 *Cx.pipiens* form *molestus*, 3 *pipiens* /*molestus* hybrids and 23 *Cx. pipiens* mosquitoes with “unknown status” (1 from Greece, Cyprus with negative result after CQ11 assay, and 22 *Cx. pipiens* mosquitoes from Italy and Israel whose status was not determined by CQ11 assay) (Table 4). Within the 544 specimens with known taxonomic status (580 minus 36 “unknown” status (Table 1), a strong association was observed between *COI* haplotype or group and taxa, being group A more frequent in *Cx. pipiens* form *pipiens* (91 %), type D in *Cx. pipiens* form *molestus* (81 %), and group E in *Cx.quinquefasciatus* (42 %) and in its hybrids with *Cx. pipiens* (32 %) (Chi-square = 732.71, d.f. = 8, *P* < 0.0001).

### Phylogenetic analysis

**Fig. 2:**
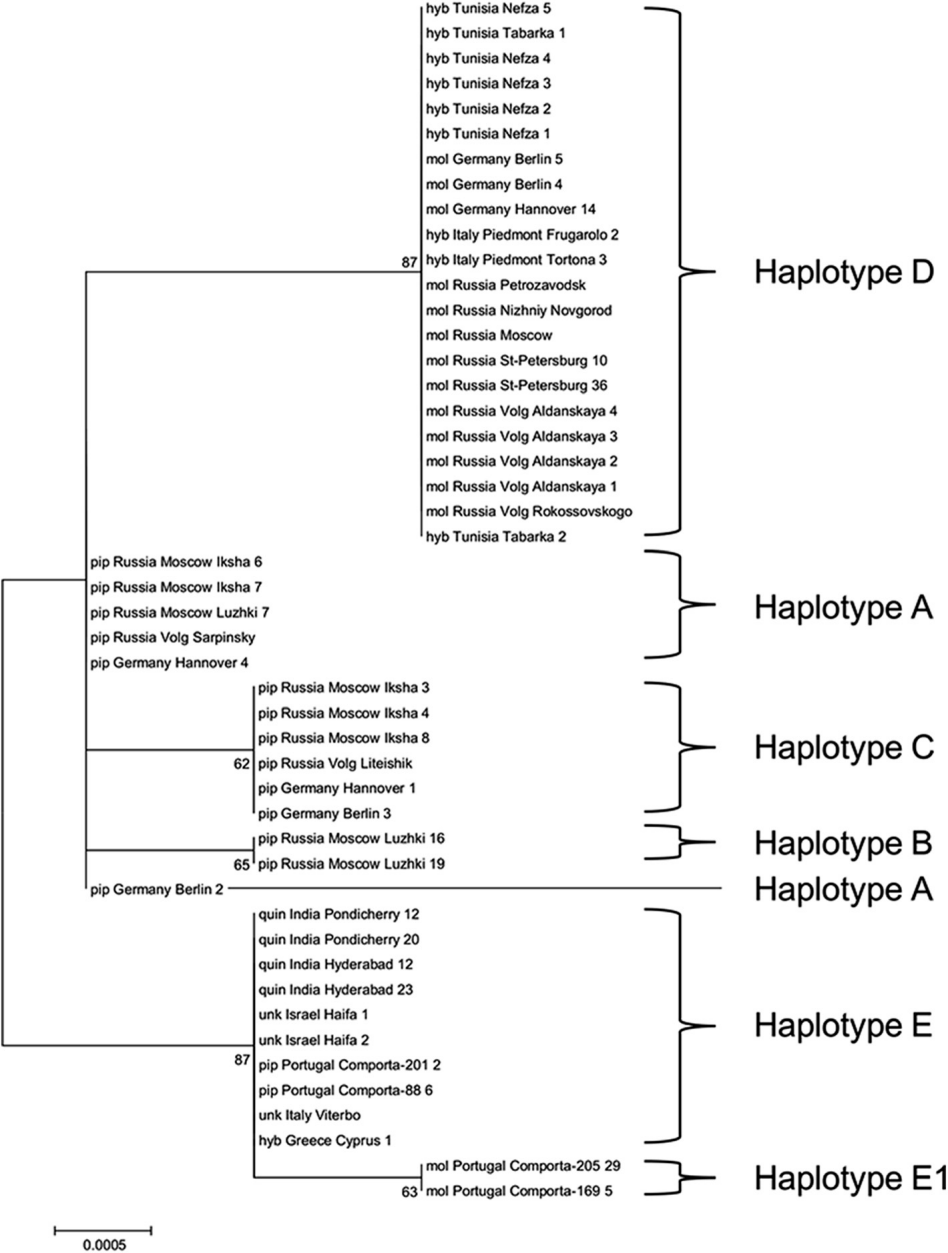
Molecular Phylogenetic analysis of *COI* gene from *Cx. pipiens* taxa. A total of 48 sequences within the 54 mentioned in Table 2 were analyzed. The evolutionary history was inferred by using the Maximum Likelihood method based on the Hasegawa-Kishino-Yano model [46]. The tree with the highest log likelihood (−1539.7457) is shown. Initial tree(s) for the heuristic search were obtained by applying the Neighbor-Joining method to a matrix of pairwise distances estimated using the Maximum Composite Likelihood (MCL) approach. The tree is drawn to scale, with branch lengths measured in the number of substitutions per site, numbers are bootstrap coefficients calculated for a 1000 repeats. All positions containing gaps and missing data were eliminated. There were a total of 1150 positions in the final dataset. Evolutionary analyses were conducted in MEGA6 [44]. Taxa names: pip – *Cx. pipiens* form *pipiens*, mol - *Cx. pipiens* form *molestus*, quin - *Cx. quinquefasciatus*, hyb – hybrid, unk – unknown or *Cx. pipiens* sl

Molecular Phylogenetic analysis of 48 sequences with 1150 bp of *COI* gene from *Cx. pipiens* taxa, was carried out using the Maximum Likelihood method based on the Hasegawa-Kishino-Yano model (Fig. 2). The average total A + T content was 70.4 % and both variable or Parsimony informative sites were 0.006 % (7/1150). The tree topology supports the data from our RFLP analysis. However, it does not confirm a phylogenetic relation between taxa groups, as the bootstrap values uniting the different groups are quite low (<75), with the exception of haplotype D and haplotypes E and E1 with bootstrap values of 87.

## Discussion

Three mosquito members of the *Culex pipiens* complex (*Cx.pipiens* form *pipiens*, *Cx.pipiens* form *molestus* and *Cx.quinquefasciatus*) were sampled for mtDNA study. All known studies on *COI* gene polymorphism in *Cx. pipiens* complex mosquitoes investigated only the 5′ half of the gene, the so-called Barcode sequence, while we studied a larger size segment (1150 bp) spanning almost the whole gene sequence (1536 bp). Variability in *COI* gene is very low, but nevertheless, fixed nucleotide substitutions that can distinguish the complex members in allopatric populations were found. Statistically significant correlation between *COI* haplogroup/type and taxa has been observed also in sympatric populations after the confirmation of taxonomy status by CQ11 assay (Tables 3 and 4). It has been shown that CQ11 assay can produce misleading results in identification of *Culex pipiens* complex members and should not be the single method to be used [26]. Discrepancies of the CQ11 results with taxonomy status of *Cx. pipiens* have been identified in other studies [47, 48]. Although many authors made conclusions based on the use of only one marker - CQ11 [21, 23] we used this method in combination with other nuclear marker *ACE2* and two cytoplasmic markers - *COI* and *w*Pip. The use of these markers in conjunction has enabled us not only to clarify the taxonomic status of the individual, but also to discover hybrids between *Cx. pipiens* and *Cx. quinquefasciatus* previously unknown to the Mediterranean region, which were confirmed by sequencing of *ACE*2 gene and finding individuals with both alleles*.*

**Fig. 3:**
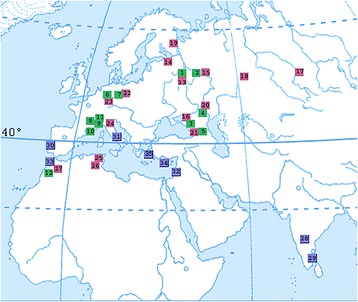
Geographic distribution of *COI* haplotypes. Numbers in the map correspond to locality numbers in Table 1; green, *COI* types A-C; pink, *COI* type D*;* blue, *COI* types E and E1

Six distinct haplotypes, based on *COI* sequences were observed. However, since we did not sequence fully all samples, which would have been incompatible with our constraints, we cannot exclude the possible existence of other haplotypes, although we are comfortable that the amount of sequences we studied is representative of our sample. The distribution of this mtDNA diversity appeared to have some degree of spatial structure as mtDNA haplogroup A and haplotype D occured both in northern European and Mediterranean populations, whereas haplogroup E was found exclusively in Mediterranean and Indian populations (Fig. 3). E/E1 were the only haplotypes to be found in Portugal, on the Greek islands, in Israel and in India. In Israel, this low variability can be attributed to the low sampling (a single locality and small sample size, *N* = 7), whereas in Greece and India, at least two localities (27 and 43 samples respectively), were investigated which does not preclude the possible existence of other haplotypes.

Haplotype A has also been recently found to be common in *Cx.pipiens* form *pipiens* from open habitats in England [26], southern Germany [27] and in other populations from Russia [38]. More polymorphism of *COI* in *Cx. pipiens* form *pipiens* was found in study of large number of sequences in Germany [49]. Most of sequences (361 from 399) correspond to our haplotype *COI*-A, others differ from it in some additional SNP. Haplogroup A is thus dominant in above ground populations in European countries with temperate climates. However, haplotype A was found in mosquitoes from the laboratory line T7 from Montpellier, France. This line descended from an underground population with autogeny (O. Duron, personal communication) and consists of *molestus* and hybrid according to the CQ11 assay (Table 3).

Haplotype D is commonly found in *molestus* populations from Russia, as well as from Germany in a total of 7 populations (Tables 1 and 3). It has also been detected in France and Serbia [27], as well as England [26] and in laboratory colony in Turkey [48]. The identical *COI* nucleotide sequences found in *Cx.pipiens* form *molestus* from such different locations, furthermore supported by the phylogenetic analysis, favors the assumption of a unique mutation in this gene and the subsequent migration of mosquitoes, rather than the hypothesis of *molestus* populations arising from neighboring, or sympatric, *pipiens* populations in northern European countries [50]. The haplotype D was found also in specimens from open above ground habitats in Italy, Tunisia, and Morocco, in a total of 4 populations. In the latter cases, the ovarian status of females (autogeny/anautogeny) was not determined and the taxonomic status of these specimens based on CQ11 assay varies: they are characterized as *pipiens* or *molestus* forms, and hybrids (Table 3). All individuals with haplotype D were infected with *w*PipIV. The presence of hybrids in the southern populations is consistent with the hypothesis of less reproductive isolation between *pipiens* and *molestus* forms in southern latitudes compared to the northern region [1, 20]. There has been evidence that gene flow between the two forms of *Cx. pipiens* is limited in Russia [38], Germany [51] and the US [52]. Nevertheless, using microsatellite analyses hybrids between *pipiens* and *molestus* in Palearctic have been detected in Portugal [20], Greece [24], Germany [53] and by CQ11 assay in Morocco [23]. However, the percentage of hybrid individuals in the northern countries was much less than in the southern. Hybrids between *pipiens* and *molestus* have also been found in the US, but there is sufficient evidence that the US *molestus* genetically differs from European [54]. Hybridization is probably responsible for the absence of any strict correlation between *COI* haplotype and taxa within the complex.

Two polymorphic haplotypes, that we grouped to haplogroup E were detected in *Cx. quinquefasciatus* from two laboratory strains from India and in 7 *Cx.pipiens* populations from Mediterranean countries, either of form *pipiens*, *molestus,* mixed or unknown based on CQ11 assay. Remarkably, the mtDNA haplotype E is rarely found in temperate climates: the northernmost point at which it was observed is the Viterbo population in Italy. Furthermore, haplotype E1was found only in Portugal, the westernmost location in Europe. These two haplotypes, E and E1, have been found in both anautogenous and autogenous Portuguese mosquitoes, but E1 was more frequent in the *molestus* form (0.86) than in the *pipiens* form (0.14) (Table 2). Individuals with haplotypes Е and Е1were infected with *w*PipI.

While these haplotypes were detected in *Cx.pipiens* specimens, GenBank data shows that haplotype E is commonly observed in *Cx. quinquefasciatus* specimens from various tropical and subtropical countries: Uganda: GQ165791, GQ165796 and GQ165798; Iran: JQ958373 and FJ210909; Thailand: HQ398883; India: AY729977, DQ267689 and EU259297; Brazil: GQ255650; South Africa: GU188856. Haplotype E and another, that differs from E by one additional SNP have been recently found in populations of *Cx. quinquefasciatus* in Malaysia, although in some specimens was found haplotype A [55]. Sequences, identical to haplotype E have also been recently found in *Cx. quinquefasciatus* in Southern Turkey [56].

According to data in GenBank, the presence of other haplotypes, from group A and type D, in *Cx. quinquefasciatus* from Africa was not observed. Simultaneously, all studied 245 *Cx. quinquefasciatus* individuals from 19 allopatric populations of sub-Saharan Africa from 12 countries are characterized by *w*PipI infection [35]. Since in this work, a strong association was observed between *COI* haplotype and *Wolbachia* type, further studies would be able to bring to evidence if *quinquefasciatus* from sub-Saharan Africa would also have just haplotype E, or others and what variants.

With the exception of *Cx. quinquefasciatus* from India and “*pipiens*/*quinquefasciatus*” hybrid specimens from Kos (Greece) and Tanger (Morocco), all individuals studied in this work harboring *COI* haplotypes E and E1, as well as *w*Pip type I infection, were deemed *Cx. pipiens,* according to ACE2 assay, and probably represent examples of cytoplasmic introgression, which was also observed in other members of this mosquito complex [35].

There are many examples of the introduction of *Cx. quinquefasciatus* worldwide through commercial, air or sea traffic [e.g. 57]. Typically, introduced *Cx. quinquefasciatus* initially appears in seaports, spreads along coastal areas and eventually moves inland following human activity [50]. *Cx. pipiens* with E and E1 haplotypes were also found in coastal areas of Italy, Portugal, Greece, Israel and Morocco (Fig. 3). Random importation of *Cx. quinquefasciatus* and crossing with local *Cx. pipiens* may have resulted in the generation of hybrid populations, as we discovered on the Greek island of Kos [13] and in Tanger, Morocco (this work). Such is the case in the hybrid zone between the 30° and 40° parallels in North America [5, 7, 8, 14], and in Argentina [10] proving the absence of crossing barriers between *Cx. quinquefasciatus* and *Cx. pipiens*. Thereafter, hybrid individuals may have backcrossed with local *Cx. pipiens* mosquitoes and after several generations, there are both hybrids’ nuclear genome as well as *Cx.pipiens* DNA. Therefore, we find individuals with *Cx.pipiens’* nuclear genetic background while having *Cx. quinquefasciatus’* maternal mtDNA.

Molecular Phylogenetic analysis of 48 sequences of *COI* gene from *Cx. pipiens* s.l. taxa yielded a tree topology that supports the data from the RFLP analysis. However, its bootstrap values were only significant between haplotypes E/E1 and D. Despite the fact that a strong association was observed between *COI* haplotype and taxa, the variability in *COI* gene is low, also evidenced by the low Parsimony informative sites, already detected in members of *Cx. pipiens* s.l. [40], therefore *COI* gene may not be the better marker to infer the evolutionary relationship of such close taxa and more polymorphic markers or a multilocus analysis might be more informative. However, higher variability was detected when *Cx. torrentium* and *Cx. pipiens* s.l. were compared [40]. On the other hand, absence of significant differences within polymorphic haplogroups A and E, may indicate their evolutionary proximity and that mutations, distinguishing these haplotypes, occurred after the divergence of lines infected with a certain type of bacteria. Further studies on larger samples may also shed light on this issue.

*Wolbachia pipientis* types I, II and IV were found in this study and in association with particular *COI* haplotypes/groups. In contrast, *w*PipIII and *w*PipV groups were not detected in the investigated samples. *w*PipV is spread in Southeast Asia [35], from where we had no specimens, while*w*Pip III is widespread in Western Europe and the Americas. It is possible that this variant was not found in this study due to the relatively small sample size of mosquito collections from Western Europe.

The association between the *COI* haplotype and the group of cytoplasmically transmitted symbiotic intracellular bacterium *Wolbachia* shows the co-transmission of cytoplasmic components and cytoplasmic introgression that appears to occur frequently between *Cx.pipiens* members in the Mediterranean region: all *COI* haplotypes and hybrids based on ACE2 and CQ11 assays were found in specimens from most tested countries. Previous investigations examining either microsatellites [20] or the mtDNA *cytb* gene and *Wolbachia* polymorphism [35] also support frequent hybridization events within the *Cx.pipiens* complex in the Mediterranean Basin. Thus, by contrast with Northern Europe, similar mtDNA haplotypes are found in different *Cx.pipiens* taxa in the Mediterranean region. Our understanding of the contribution of cytoplasmic inheritance remains inadequate. Mosquitoes with hybrid features may vary from the parental forms in their competence for the transmission of pathogens. The absence of *Cx. pipiens* with haplotypes from group E in northern temperate climates could hypothetically point to different population origins, with possible interference in their physiology, of which they could be an indicator. Mosquitoes with haplotypes from group E may have obtained cytoplasmic genes from *Cx. quinquefasciatus* that feeds readily on birds and mammals and is an extremely efficient vector of encephalitis viruses, including West Nile Virus, Rift Valley Fever Virus, and is also responsible for transmitting the filarial nematode, *Wuchereria bancrofti* (Cosmotropical areas) [58] and is therefore more medically important than *Cx. pipiens*. A catholic feeding behavior of hybrids has often been associated with a higher potential for transmitting arbovirus [6]. The evidence presented in this paper requires further study to concomitantly examine the genetic structure of the population, the associations with biologically important behaviors and the vector competence of *Cx. pipiens* from different populations and from various areas in the Mediterranean region and in other regions where distribution of complex members can overlap.

## Conclusions

The data from this study confirm the lack of reproductive barriers in *Cx. pipiens* forms *pipiens* and *molestus* natural populations in southern European countries, compared with the northern. Hybrids between *Cx. pipiens* and *Cx. quinquefasciatus* were reported in the Americas and India. In the coastal areas of the Mediterranean we found hybrids between endemic *Cx. pipiens* and *Cx. quinquefasciatus*, that were likely introduced. These cases of hybridization can change the properties of vectors due to the genetic contribution of the more antropophylic *Cx. quinquefasciatus.* The analysis of the genetic structure of mosquito populations sheds light on the processes taking place, increasing the understanding of the epidemiology of the diseases these mosquitoes transmit, essential for the improvement of prevention and control policies of these diseases. The relevance of these findings is heightened in a context of climate changes and introduction of exotic vectors and (re)-emerging diseases.

## Additional files

Additional file 1:
**Discrimination of specific** ***COI*** **alleles.** (A) *COI* haplotypes after *Hae*III digestion of PCR products: 1-type A, 2-type B, 3-type C, 4-type D, 5-type E, 6-type E1, 7-marker molecular weight M100; (B) *COI* haplotypes after *Alu*I digestion: 1-type A, 2-type B, 3-type C, 4-type D, 5-type E, 6-type E1, 7-marker molecular weight M50. (TIF 141 kb) Additional file 2:
**Example of PCR amplification of specific ACE2 (A) and CQ11 (B) alleles in Tanger, Morocco.** 1–13 - samples, samples 7, 10 - *Cx. pipiens* form *pipiens* by both assay. Other samples are hybrids by ACE2 or CQ11 assays; 14 - marker molecular weight; 15 – *Cx. quinquefasciatus*; 16 – *Cx.pipiens. (TIF 1408 kb)*
Additional file 3:
**Alignments of** ***ace-2*** **gene sequences for** ***Cx. pipiens/quinquefasciatus*** **hybrid collected from Kos, Greece.** Sequences are compared with *Cx. pipiens* (AY196910) and *Cx. quinquefasciatus* (AY196911).“*” Indicates the absence of mutation, “.” - nucleotide substitutions, “-” indels. (DOCX 12 kb) Additional file 4:
**Discrimination of specific** ***w*** **Pip alleles based on** ***ank2*** **and** ***pk1*** **markers.** (A) three alleles: a (313 bp), b (217, 195, 98 bp) and c (293, 217 bp) after *Hinf*I digestion of the *ank2* PCR products; (B) three alleles: a/e (903, 430 bp), c (851, 498 bp) and d (497, 251, 107 bp) after *Taq*I digestion of the *pk1* PCR products; (C) allele a (903, 303, 141 bp) after *PstI* digestion of the *pk1* PCR products. (TIF 557 kb)
