# Supplementary figures and images for: Genetic diversity of Culex pipiens mosquitoes in distinct populations from Europe: contribution of Cx. quinquefasciatus in Mediterranean populations
Source: Parasit Vectors. 2016 Jan 27;9:47. doi: 10.1186/s13071-016-1333-8 (PMC4730663; doi:10.1186/s13071-016-1333-8)

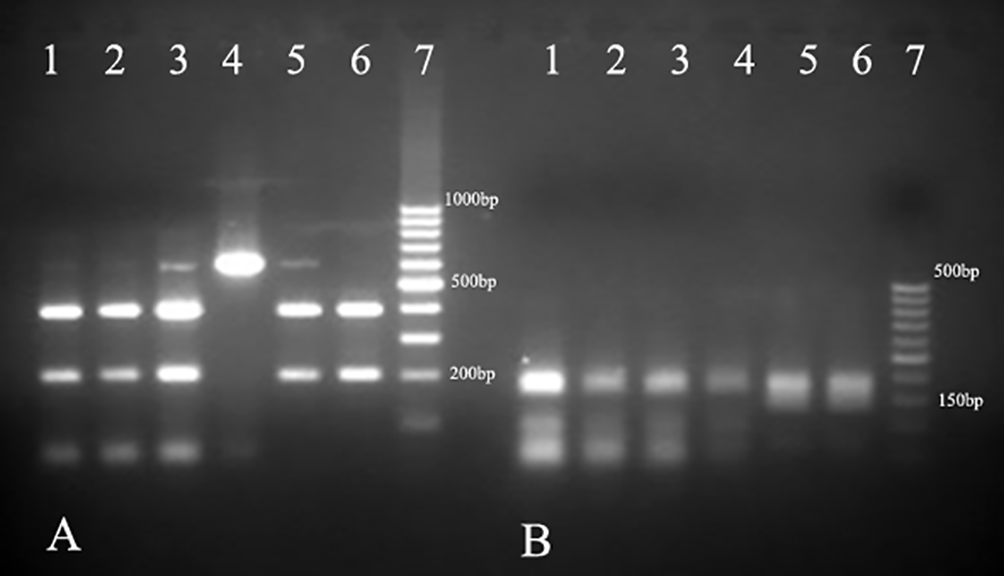

Supplement: Additional file 1: — Discrimination of specific COI alleles. (A) COI haplotypes after HaeIII digestion of PCR products: 1-type A, 2-type B, 3-type C, 4-type D, 5-type E, 6-type E1, 7-marker molecular weight M100; (B) COI haplotypes after AluI digestion: 1-type A, 2-type B, 3-type C, 4-type D, 5-type E, 6-type E1, 7-marker molecular weight M50. (TIF 141 kb) [file 13071_2016_1333_MOESM1_ESM.tif]

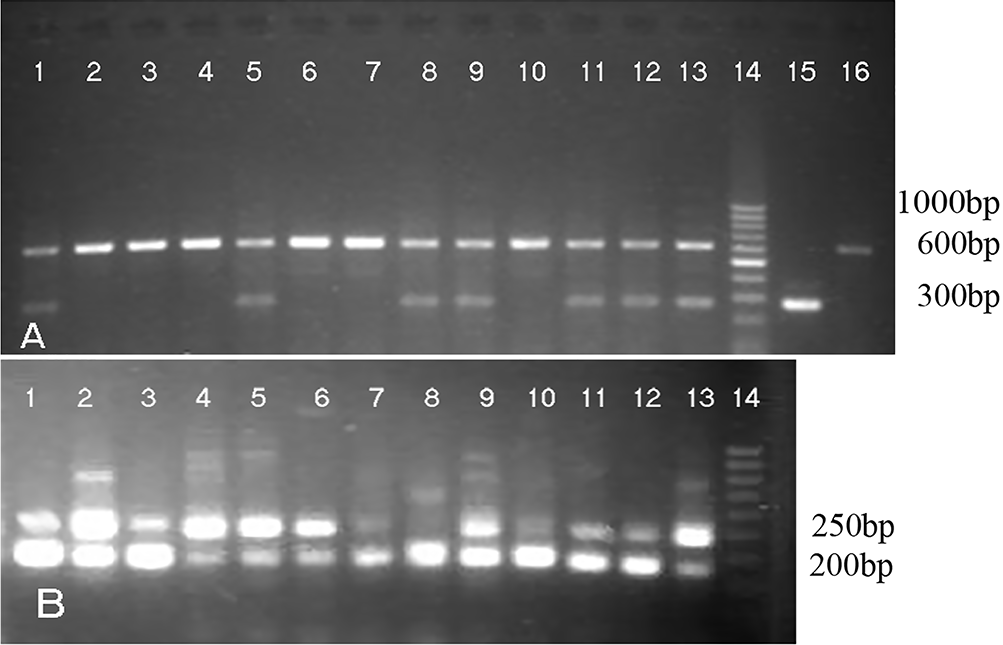

Supplement: Additional file 2: — Example of PCR amplification of specific ACE2 (A) and CQ11 (B) alleles in Tanger, Morocco. 1–13 - samples, samples 7, 10 - Cx. pipiens form pipiens by both assay. Other samples are hybrids by ACE2 or CQ11 assays; 14 - marker molecular weight; 15 – Cx. quinquefasciatus; 16 – Cx.pipiens. (TIF 1408 kb) [file 13071_2016_1333_MOESM2_ESM.tif]

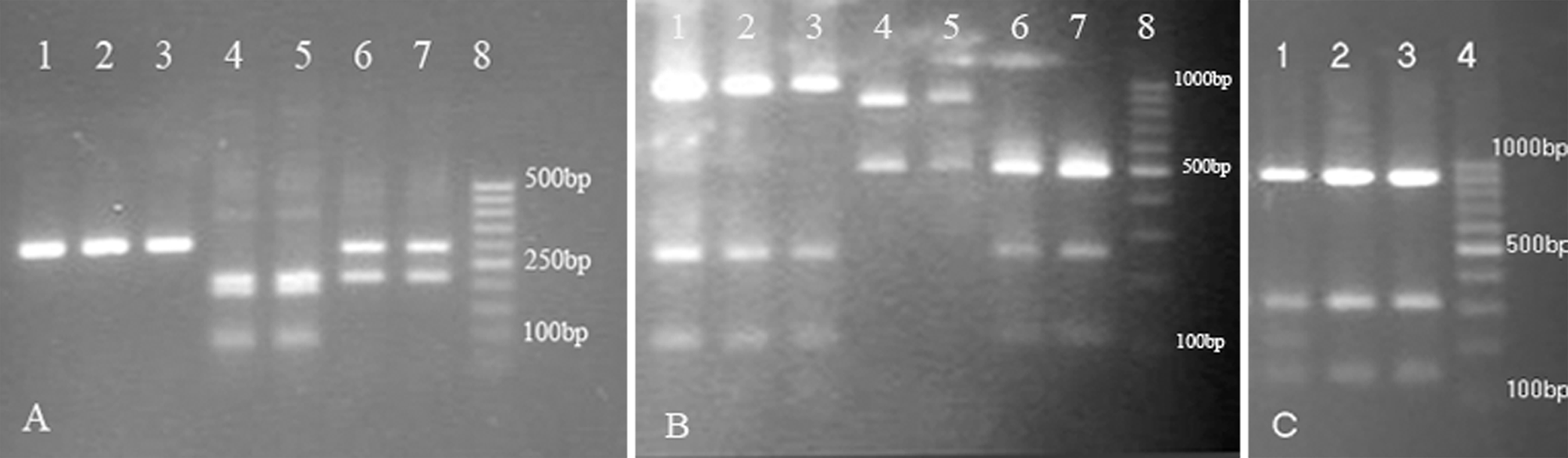

Supplement: Additional file 4: — Discrimination of specific w Pip alleles based on ank2 and pk1 markers. (A) three alleles: a (313 bp), b (217, 195, 98 bp) and c (293, 217 bp) after HinfI digestion of the ank2 PCR products; (B) three alleles: a/e (903, 430 bp), c (851, 498 bp) and d (497, 251, 107 bp) after TaqI digestion of the pk1 PCR products; (C) allele a (903, 303, 141 bp) after PstI digestion of the pk1 PCR products. (TIF 557 kb) [file 13071_2016_1333_MOESM4_ESM.tif]
