# Supplementary material for: Genetic diversity of Culex pipiens mosquitoes in distinct populations from Europe: contribution of Cx. quinquefasciatus in Mediterranean populations
Source: Parasit Vectors. 2016 Jan 27;9:47. doi: 10.1186/s13071-016-1333-8 (PMC4730663; doi:10.1186/s13071-016-1333-8)
Supplement: Additional file 3: — Alignments of ace-2 gene sequences for Cx. pipiens/quinquefasciatus hybrid collected from Kos, Greece. Sequences are compared with Cx. pipiens (AY196910) and Cx. quinquefasciatus (AY196911).“*” Indicates the absence of mutation, “.” - nucleotide substitutions, “-” indels. (DOCX 12 kb) [file 13071_2016_1333_MOESM3_ESM.docx]

Additional file 4. **Alignments of *ace-2* gene sequences for *Cx. pipiens/quinquefasciatus* hybrid collected from Kos, Greece.**

#Cx.pipiens CTGTCTGTAT CTGAACATTT GGGTACCAAC GAAAACCCGT TTGCGCCACG GACGAGGACT AAACTTTGGA AACAACGACG TATGTACTAC TTCTTCTTGT [100]

#Kos1_clon1 .......... .......... .......... .......... .......... .......... .......... .......... .......... .......... [100]

#Kos1_clon2 .......... .......... .......... ...G...... ........T. .......... .......... ........T. ..A....... .......... [100]

#Cx.quinq .......... .......... .......... ...G...... ........T. .......... .......... ........T. ..A....... .......... [100]

#Cx.pipiens TAGTACACAG TACGACAGAA AAATGTCGAT GATGGCTCTG TCAGAAGTTT TTTGACAATC ACTTTTTGAT TCTTCGATCA TTCGGAAGAA TTTATAGTGA [200]

#Kos1_clon1 .......... .......... .......... .......... .......... .......... .......... .......... .......... .......... [200]

#Kos1_clon2 .......... A...C..... ....A..... ....-..... .T..G.T... .......... G...A...G. ........G. ....A.G... ........A. [200]

#Cx.quinq .......... A...C..... ....A..... ....-..... .T..G.T... ....G..... G...A...G. ........G. ....A.G... ........A. [200]

#Cx.pipiens TATGGTGGAA ACGCATGATA CCAGATATGA GAACTAACTA AACTTTTGAC ATTTTTCTGT CGAGCTGTGC TTGTGATGAT TTAGTTGTTC GTGGCTCTGA [300]

#Kos1_clon1 .......... .......... .......... .......... .......... .......... .......... .......... .......... .......... [300]

#Kos1_clon2 A.....T..G .......... .T.A...... .........G .......A.A .......... .......... .....G.... .......... .C........ [300]

#Cx.quinq A.....T..G .......... .T.A...... .........G .......A.A .......... .......... .....G.... ........G. .C........ [300]

#Cx.pipiens GAGAGACAGG TCCAGAGTGT ATTTTTTAGT AGTTGCGTAG GCGTTTATGC ACCCACAACG GAGATAATTC ACAAGG-TTT TTTTTCTTTT CTTTTTGTTT [400]

#Kos1_clon1 .......... .......... .......... .......... .......... .......... .......... ......-... .......... .......... [400]

#Kos1_clon2 .......... .......... .......... ...A...... .......... ........A. .......... ......-... .......... ...---.... [400]

#Cx.quinq .......... .......... .......... ...A...... .......... ........A. .......... ......T... .......... ...---.... [400]

#Cx.pipiens TTTCCCCTCT GGCATGGCCG TGGCCACCTC TTTATTGCAG TACTTCCAGG ACGATGATGA CTTCCAGCGG CAGCACCAGT CCAAGGGCGG CCTCGCGATG [500]

#Kos1_clon1 .......... .......... .......... .......... .......... .......... .......... .......... .......... .......... [500]

#Kos1_clon2 ......T... T.A.....T. ....A..... .......... .......... .......G.. .......... .......... .......... .......... [500]

#Cx.quinq ......T... T.A.....T. ....A..... .......... .......... .......G.. .......... .......... .......... .......... [500]

#Cx.pipiens CTGGTCTGGA TCTACGGGGG TGGGTTTATG AGCGGAACAT CAACGTTGGA CGTTTACAAC GCAGAAATAC TGGCGGCCGT TGGAAACGTA ATCGTGGCCT [600]

#Kos1_clon1 .......... .......... .......... .......... .......... .......... .......... .......... .......... .......... [600]

#Kos1_clon2 .......... .......... .......... .......... .....C.... .......... .......... .......... .......... .......... [600]

#Cx.quinq .......... .......... .......... .......... .....C.... .......... .......... .......... .......... .......... [600]

#Cx.pipiens CGATGCAGTA CCGAGTGGGA GCATTCGGTT TCTTCTACCT [640]

#Kos1_clon1 .......... .......... .......... .......... [640]

#Kos1_clon2 .......... .......... .......... .......... [640]

#Cx.quinq .......... .......... .......... .......... [640]

Sequences are compared with *Cx. pipiens* (AY196910) and *Cx. quinquefasciatus* (AY196911).“.” Indicates the absence of mutation, "-" indels. Alignment was done in the MEGA 6 [44].
